# Temperature-Triggered/Switchable Thermal Conductivity of Epoxy Resins

**DOI:** 10.3390/polym13010065

**Published:** 2020-12-26

**Authors:** Matthias Sebastian Windberger, Evgenia Dimitriou, Sarah Rendl, Karin Wewerka, Frank Wiesbrock

**Affiliations:** 1 Polymer Competence Center Leoben GmbH, Roseggerstrasse 12, 8700 Leoben, Austria; matthias.windberger@pccl.at (M.S.W.); evgeniaod@auth.gr (E.D.); sarah.rendl@fh-wels.at (S.R.); 2 Institute for Chemistry and Technology of Materials, Graz University of Technology, NAWI Graz, Stremayrgasse 9, 8010 Graz, Austria; 3 Institute for Electron Microscopy and Nanoanalysis and Center for Electron Microscopy, Graz University of Technology, NAWI Graz, Steyrergasse 17, 8010 Graz, Austria; karin.wewerka@felmi-zfe.at

**Keywords:** epoxy resin, crosslinked polymer, thermal conductivity, inorganic filler, π-π stacking, polymer from natural resources, bisphenol-free epoxy resin

## Abstract

The pronouncedly low thermal conductivity of polymers in the range of 0.1–0.2 W m^−1^ K^−1^ is a limiting factor for their application as an insulating layer in microelectronics that exhibit continuously higher power-to-volume ratios. Two strategies can be applied to increase the thermal conductivity of polymers; that is, compounding with thermally conductive inorganic materials as well as blending with aromatic units arranged by the principle of π-π stacking. In this study, both strategies were investigated and compared on the example of epoxy-amine resins of bisphenol A diglycidyl ether (BADGE) and 1,2,7,8-diepoxyoctane (DEO), respectively. These two diepoxy compounds were cured with mixtures of the diamines isophorone diamine (IPDA) and *o*-dianisidine (DAN). The epoxy-amine resins were cured without filler and with 5 wt.-% of SiO_2_ nanoparticles. Enhanced thermal conductivity in the range of 0.4 W·m^−1^·K^−1^ was observed exclusively in DEO-based polymer networks that were cured with DAN (and do not contain SiO_2_ fillers). This observation is argued to originate from π-π stacking of the aromatic units of DAN enabled by the higher flexibility of the aliphatic carbon chain of DEO compared with that of BADGE. The enhanced thermal conductivity occurs only at temperatures above the glass-transition point and only if no inorganic fillers, which disrupt the π-π stacking of the aromatic groups, are present. In summary, it can be argued that the bisphenol-free epoxy-amine resin with an epoxy compound derivable from natural resources shows favorably higher thermal conductivity in comparison with the petrol-based bisphenol-based epoxy/amine resins.

## 1. Introduction

The ongoing miniaturization, in particular in the field of microelectronics, is the key prerequisite for the development and introduction-to-market of devices with continuously enhanced power-to-volume ratios [1,2,3]. Because of this enhanced power-to-volume ratio, nonetheless, correspondingly increased (maximum) temperatures during operation of the devices must be considered, which have an effect on all of the components of the devices. Considering, for example, a multilayer assembly composed of alternating layers of metals/semiconductors and polymers/polymer-based composites, both thermal conductivity and thermal expansion have to be considered [4,5,6]. The coefficient of thermal expansion of metals and semiconductors is commonly in the range of/lower than 20 ppm·K^−1^ (e.g., α (copper) = 16.6 ppm·K^−1^ [7]; α (silicon) = 2.6 ppm·K^−1^ [8]; α (aluminum) = 22.9 ppm·K^−1^ [7]), while that of polymers spans the common range from 50 to 200 ppm·K^−1^ (e.g., α (epoxy resin) = 80 ppm·K^−1^ [9], α (polyamide) = 158 ppm·K^−1^ [10], α (unfilled polypropylene) = 140 ppm·K^−1^ [11]). Hence, in a multilayer assembly alternatingly composed of (conducting) layers of copper and (insulating) layers of epoxy resin, the coefficients of thermal expansion differ by a factor of 5 and, consequently, delamination of the polymer layers and subsequent device failure are likely to occur at elevated temperatures.

One strategy to influence (lower) the coefficient of thermal expansion of polymers is filling with inorganic particles, particularly reinforcement with fibers. Very few inorganic fillers with a negative coefficient of thermal expansion have been reported [12]. Glass-fiber reinforced polypropylene, e.g., shows coefficients of thermal expansion as low as 20 ppm·K^−1^ [13], in contrast to that of unfilled polypropylene in the range of 140 ppm·K^−1^ [11]. The filling of polymers with inorganic particles and/or fibers deteriorates the recyclability of such composites and, furthermore, significantly alters the mechanical properties of the composites (in comparison with the unfilled polymers).

Alternatively, the thermal conductivity of polymers can be (primarily) addressed. The thermal conductivity of metals/semiconductors is commonly higher than 100 W·m^−1^·K^−1^ (e.g., λ (copper) = 429 W·m^−1^·K^−1^, λ (silicon) = 148 W·m^−1^·K^−1^, λ (aluminum) = 237 W·m^−1^·K^−1^) [14] and, as such, significantly higher than that of (unfilled) polymers, which commonly exhibit thermal conductivity lower than 0.2 W·m^−1^·K^−1^ [15,16]. Correspondingly, polymers in multi-layer assemblies are likely to act as ‘heat sinks’ because of their short-coming in heat dissipation—the temperature of the polymers increases to unfavorably high values and, in addition to the thermal expansion, polymer degradation is likely to occur as well.

Two main strategies have been reported for the enhancement of the thermal conductivity of polymers and polymer-based composites; that is, compounding with inorganic particles that exhibit high thermal conductivity (e.g., λ (BN) = 600 W·m^−1^·K^−1^ [17] λ (SiO_2_) = 0.7 W·m^−1^·K^−1^ [18]) on the one hand, and blending with organic moieties with a high content of aromatic units capable of forming (crystalline) regions by π-π-stacking on the other [19,20,21]. The so-called percolation threshold [22] has to be considered within both strategies, which commonly results in high degrees of compounding/blending and influences various other macroscopic properties such as the mechanical properties. Within the compounding strategy, the unfavorably high pricing of boron nitride has to be considered as well [23,24].

**Figure 1:**
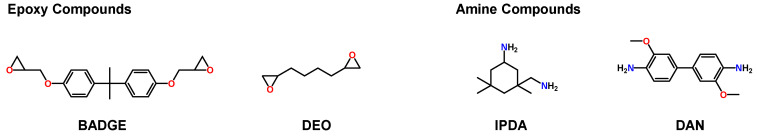
Chemical structures of the diepoxy compounds BADGE (bisphenol A diglycidyl ether) and DEO (1,2,7,8-diepoxyoctane) as well as the diamine compounds IPDA (isophorone diamine) and DAN (*o*-dianisidine).

Hence, in this study, the effects of compounding polymers with (low amounts of) silica particles and the blending of polymers with aromatic rich monomers were investigated on the example of epoxy resins. Meeting the scientific research interest in and the popular demand for polymers from renewable resources, which have manifested themselves in scientific research efforts in that field, monomers for the production of bio-based polymers [25] were considered in this study as well. In addition to the (comparably less flexible) monomer bisphenol A diglycidyl ether (BADGE), 1,2,7,8-diepoxyoctane (DEO) was also investigated as a diepoxy compound (Figure 1). Notably, the non-aromatic aliphatic diepoxy compound DEO can be derived from pseudo-pelletierine (contained in the bark of the pomegranate tree) [26]. These epoxy compounds were cured with two different amines, isophorone diamine (IPDA) and *o*-dianisidine (DAN) (which differ in the content of aromatic units; Figure 1). IPDA is derived from isophorone, which naturally occurs in cranberries [27].

## 2. Materials and Methods

### 2.1. Materials

DEO (>97%) and DAN (>98%) were purchased from TCI (Eschborn, Deutschland). BADGE (97%), IPDA (>98%), and SiO_2_ nano-scaled powder (5–15 nm) were purchased from Sigma Aldrich (Vienna, Austria). All chemicals were used without further purification.

### 2.2. Instrumentation

Fourier-Transformed infrared (FT-IR) spectra were measured with a Bruker Alpha FT-IR spectrometer (Bruker Optics Inc., Billerica, MA, USA); the spectra were measured in a spectral range from 4000 to 400 cm^−1^, with 24 scans per sample. The attenuated total reflection (ATR) spectra were recorded using an ALPHA total internal reflection module. Differential scanning calorimetry (DSC) measurements were performed on a Mettler Toledo DSC 822e (Mettler-Toledo GmbH, Vienna, Austria) differential scanning calorimeter in order to determine the glass-transition temperatures of the unfilled epoxy resins and the nanocomposites. The samples were heated twice with a heating rate of 20 K·min^−1^ in a range of 20 to 200 °C. The thermal conductivity of the unfilled epoxy resins and the nanocomposites was quantified on a Guarded Heat Flow Meter DTC 300 (TA Instruments, New Castle, DE, USA) in the temperature range from 30 to 180 °C, namely at 30, 60, 90, 120, 150, and 180 °C. Transmission electron microscope images were conducted on a FEI Tecnai 12 transmission electron microscope (FEI, Hillsboro, OR, USA). For sample preparation, ultra-thin sections were cut from the nanocomposites using a microtome equipped with a diamond knife at room temperature. Surface energy measurements were carried out with contact angle measurements using a KRUSS DSA100 (Krüss GmbH, Hamburg, Germany) goniometer. A charge-coupled device (CCD) camera, installed on the device, was used. The method of the lying drop was used under room conditions. Bidistilled water and diiodomethane were used as solutions for determining the contact angle.

### 2.3. Analysis of the Size of the Silica Particles

**Figure 2:**
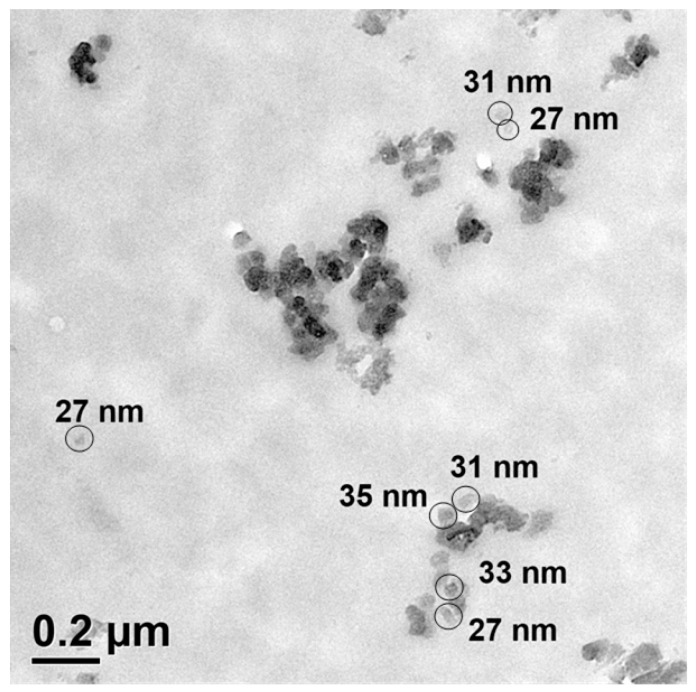
Transmission electron microscope image of silica particles dispersed in a matrix of a crosslinked copoly(2-oxazoline).

Aiming to verify the involvement of nanoparticles, the size of the commercially available silica particles was analyzed by transmission electron microscope analyses (Figure 2), for which the silica particles were dispersed in a copoly(2-oxazoline) matrix, which was crosslinked by thiol-ene click reactions [28,29,30], and cut into thin slices for analyses. The particles have a slight tendency only to agglomerate in the copoly(2-oxazoline) matrix. The transmission electron microscope images (Figure 2) reveal a size of approximately 30 nm of the silica nanoparticles, which can be recognized from the diameter analyses of individual particles within the polymer matrix.

### 2.4. Preparation of Crosslinked Epoxy Resins and Nanocomposites

One equivalent of BADGE or DEO (the term “equivalent” refers to the number of epoxy units; each of the two epoxy compounds ha *two* epoxy units) was mixed with 1 equiv. of amine hardener (the term ‘equivalent’ refers to the number of protons attached to nitrogen; each of the two diamine compounds bears two primary amine groups and, correspondingly, has *four* of such protons) in the 5 g scale. The amine hardeners were added in the following ratios: wt.-%(IPDA)/wt.-%(DAN) = 0:100, 33:67, 67:33, and 100:0. Prior to mixing, the diepoxy and diamine compounds were individually heated to 150 °C. For the nanocomposites, 5 wt.-% of SiO_2_-nanoparticles was dispersed in the epoxy compound by sonication for 5 min prior to heating at 150 °C. The diamine and diepoxy compounds were mixed in stoichiometric ratio at 150 °C. Subsequently, the reaction mixtures were transferred into circular-shaped steel templates and cured at 150 °C for 30 min. Test specimens with a diameter of 50 mm and height of approx. 2 mm were obtained.

## 3. Results

### 3.1. Preparation of the Test Specimens/Curing of the Epoxy-Amine Resins

**Figure 3:**
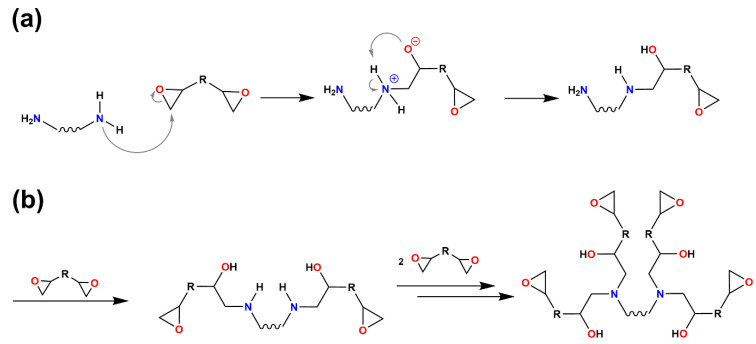
(**a**) Schematic representation of the mechanism of the epoxy-amine curing reaction. (**b**) Formation of crosslinked polymer networks.

The (targeted) enhancement of the thermal conductivity of polymer matrices by blending with aromatic units and compounding with inorganic particles was investigated on the example of epoxy-amine resins. Using ‘difunctional’ diepoxy compounds and “tetrafunctional” primary diamine compounds, the curing reaction of the polymer matrix yields crosslinked polymer networks. In order to provide identical curing conditions for all formulations [31], an unusually high curing temperature of 150 °C needed to be chosen because of the melting point of DAN. The curing reaction itself (Figure 3) involves the nucleophilic attack of the nitrogen atom on one of the α-carbon atoms of the epoxide ring, subsequent opening of the epoxide ring, and proton exchange among the ammonium group and the oxygen anion [32]. The reactivity of the (initially present) primary amine groups is higher than that of the secondary amine groups formed in the course of the epoxy-amine curing reaction. In fact, the reaction rates of the primary amine k_1_ and the secondary amine k_2_ are correlated by a ratio of k_1_/k_2_ = 1.2–1.5 [33,34,35].

For the establishment of structure–property relationships for these epoxy-amine compounds (with a focus on the thermal conductivity), a material library was synthesized, in which the diepoxy compound (either BADGE or DEO, factor: 2), the ratio of amine hardeners (wt.-%(IPDA)/wt.-%(DAN) = 100:0, 67:33, 33:67, 0:100; factor: 4), and the amount of inorganic fillers (0 or 5 wt.-% of SiO_2_; factor: 2) were varied. In summary, 2 × 4 × 2 = 16 epoxy-amine resins and composites were investigated.

### 3.2. Physico-Chemical Characterization of the Test Specimens

**Figure 4:**
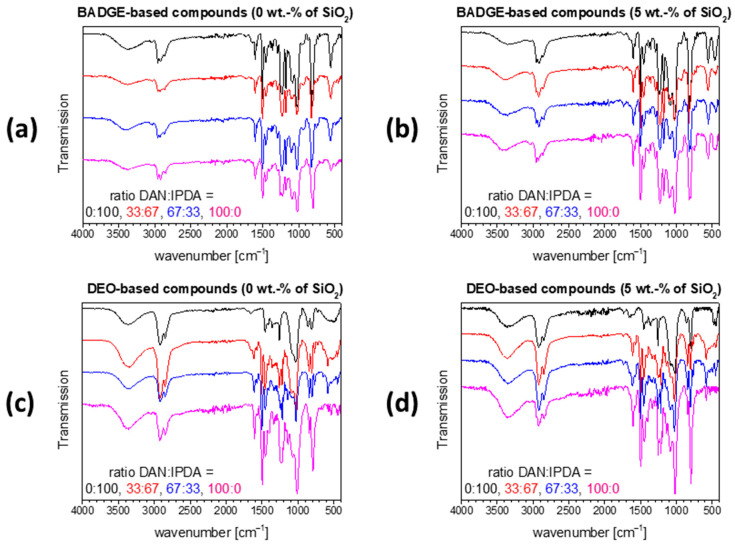
FT-IR-ATR spectra of the BADGE-based formulations without (**a**) and with silica nanofillers (**b**) as well as the DEO-based formulations without (**c**) and with silica nanofillers (**d**) after the curing reaction.

The conversion of the diepoxy and diamine monomers after the completion of the curing reaction was investigated by FT-IR-ATR spectroscopy. The IR signal characteristic for the (unreacted) epoxy ring is in the range from 810 to 890 cm^−1^. This part of the spectrum, however, is overlaid by the –CH_2_– rocking vibrations; hence, quantitative conversion of the epoxide rings (Figure 3) cannot unambiguously be argued from that signal. The signal in a range from 1085 to 1125 cm^−1^ is indicative of C-O stretching vibrations of secondary alcohols, which are not present in the reactants, but formed during the curing reactions. This signal is present in the spectra of all 16 compounds (Figure 4); it represents the successful opening of the epoxide ring.

In fully-cured epoxy-amine resins, the signals characteristic of the primary and secondary amino functions are expected to be missing. The N-H symmetrical and asymmetrical stretching vibrations in the range between 3350 and 3450 cm^−1^ as well as the N-H deformation vibrations in the range between 1500 to 1600 cm^−1^ are not visible in any of the spectra; correspondingly, quantitative curing (within the resolution of FT-IR-ATR spectra) can be argued.

The glass-transition temperatures, which can be triggered by the type of reactants as well as by the curing temperature [31], were determined by differential scanning calorimetry. Within this study, notably, the effect of alternative curing temperatures on the glass-transition temperature was not investigated; all resins and composites were cured under identical conditions in order to correlate the glass-transition points unambiguously with the molecular composition.

The eight BADGE-based compounds exhibit glass-transition temperatures in the narrow range from 146 to 154 °C. Only a minor effect of the type of diamine hardener on the glass-transition temperature is discernable; the glass-transition temperatures are only slightly lower for the DAN-rich formulations. The presence or absence of silica nanofillers does not have any effect on the respective glass-transition temperatures.

On the contrary, the glass-transition temperatures of the aliphatic DEO-based epoxy resins span a broader range of 85 to 118 °C, which is a range significantly lower than that of the aromatic BADGE-based compounds [36]. The highest glass-transition temperature of 118 °C of the DEO compounds can be observed for the silica-free DEO/IPDA resins; two effects, namely the presence of silica nanofillers and an increasing content of the DAN hardener, can be held responsible for lowering the glass-transition temperatures in the series of DEO compounds. Consequently, the DEO-based resin with the lowest/highest content of DAN and without/with silica nanofillers has the highest/lowest glass-transition temperature of 118/85 °C.

### 3.3. Thermal Conductivity of the Epoxy-Amine Blends and Composites

The thermal conductivity of the 16 test specimens was determined by Guarded Heat Flow Meter measurements. This measurement technique reveals the lowest thermal conductivity of the specimens during measurements. Preceded by studies of the thermal conductivity of composites with larger-scaled inorganic fillers, the formation of gradient composites is unlikely to occur in this study because of the exclusive involvement of nanofillers and fast curing times [18]. While π-π stacking is expected to occur exclusively at temperatures above the glass-transition temperature, the thermal conductivity was measured in a range of temperatures from 30 to 180 °C (in steps of 30 K).

**Figure 5:**
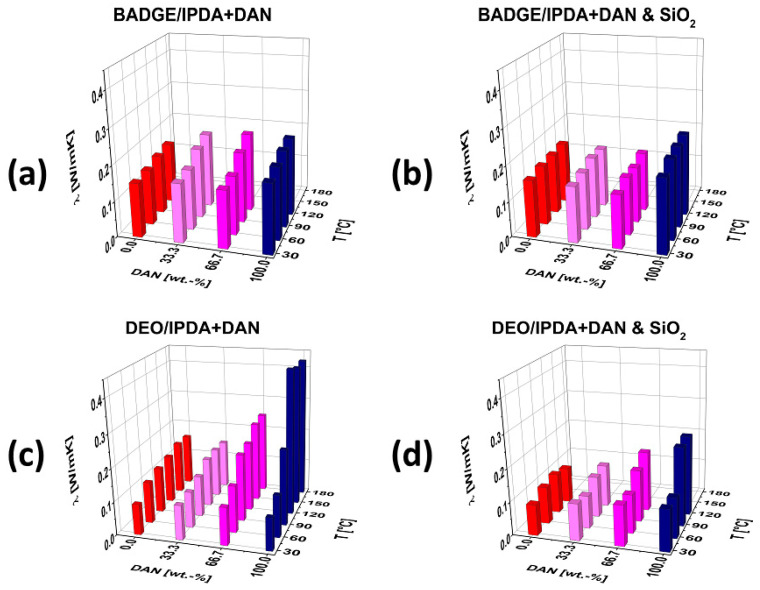
Thermal conductivity of unfilled BADGE/DEO-based epoxy-amine resins (**a,c**) and the corresponding nanocomposites with SiO_2_ fillers (**b,d**). For comparison, the Z-scales of all graphs have been drawn to the same height.

The BADGE-based blends and nanocomposites exhibit thermal conductivity in the range of 0.15 to 0.20 W·m^−1^·K^−1^ (Figure 5). For both types, the unfilled blends as well as the composites filled with silica nanoparticles, the increase of DAN content in the amine hardener mixture does not significantly enhance the thermal conductivity. In analogous fashion, the increase of the temperature above the glass-transition point has no relevant effect on the thermal conductivity of the BADGE-based resins and composites.

The measurement of the thermal conductivity of the eight DEO-containing blends and nanocomposites (Figure 5), on the contrary, displays clear trends of the variation of the thermal conductivity in the broader range from 0.08 to 0.42 W·m^−1^·K^−1^. At 30 °C, the thermal conductivity of both the unfilled resins as well as the silica nanoparticle-filled composites is in the range of 0.08 to 0.15 W·m^−1^·K^−1^, irrespective of the composition of the amine hardener mixture. The unfilled blends and the silica-containing nanocomposites that contain comparably low amounts of the amine DAN (0 and 33 wt.-%, respectively) show no significant increase of the thermal conductivity even if the temperature increases above the glass-transition point.

**Figure 6:**
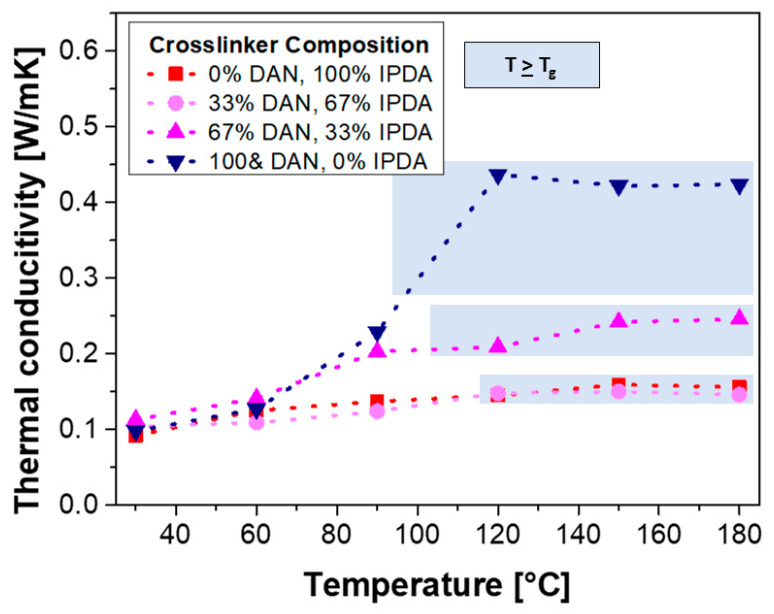
Thermal conductivity of unfilled DEO-based epoxy-amine resins as a function of the temperature; the temperatures above the glass-transition point have been highlighted in blue shade.

On the other hand, the unfilled resins with comparably high amounts of DAN (67% and 100%, respectively) exhibit significantly increased thermal conductivity in the case of elevated temperatures. Notably, the increase of the thermal conductivity is higher in the resins that were cured with DAN only and, correspondingly, contain low or no amounts of IPDA. In contrast, the silica nanoparticle-containing composites reproduce this trend to a lesser extent, which is retraced to the presence of the inorganic particles. The influence of the temperature (below/above the glass-transition point) of the unfilled DEO-based polymer networks was analyzed in detail (Figure 6).

For all DEO-based polymer networks, a slight increase of the thermal conductivity with increasing temperatures can be observed (Figure 6), which can be retraced to the molecular structure of crosslinked (co-)polymers [37]. No significant (additional) increase of the thermal conductivity at elevated temperatures above the glass-transition point can be observed for the IPDA-rich polymer networks, despite the higher chain and segment mobility (polymer networks with DAN contents of 0 and 33%, respectively). On the contrary, the polymer network formed by the involvement of only DAN shows a significant increase of the thermal conductivity from 0.229 to 0.437 W·m^−1^·K^−1^ in the temperature range from 90 to 120 °C (glass-transition temperature: T_g_ = 98 °C; Table 1); hence, it is almost doubled within this narrow range of temperatures, which can be described as ‘switchable’ thermal conductivity. This significant increase in thermal conductivity can be observed only in the DAN-rich polymer network, while high degrees of crosslinking can be argued for all polymer networks because of the quantitative occurrence of the epoxy-amine curing reaction (Figure 4), this effect can be retraced to the π-π stacking of the aromatic units of DAN.

The presence of nano-scaled silica fillers disables the significant increase of the thermal conductivity of the DEO/DAN-based polymer networks in the vicinity of the glass-transition temperature, which can be argued to originate from hindrance of the π-π-stacking of the DAN segments. On the molecular level, this hindrance is assumed to originate from interaction of the silica nanoparticles (with correspondingly large surfaces [38]) with the aromatic units of the DAN segments [39] and/or steric hindrance owing to the presence of the inorganic particles.

Hence, the DEO/DAN-based polymer network with “switchable” (temperature-dependent) thermal conductivity seems to be a potential alternative to commonly used BADGE-based epoxy/amine resins. In order to generate a first set of data for the evaluation of adhesive properties, the surface energies of the resins of this study were calculated from contact angle measurements (Table 2). For all unfilled polymer networks, an increase of the DAN content correlates with an increase of the surface energy. In greater detail, it was found that, with an increasing amount of DAN, the polar fraction of the surface energy decreases slightly, whereas the disperse fraction increases significantly. Notably, the BADGE- and DEO-based epoxy resins show comparable surface energies for a given hardener combination; the polar and disperse fractions are very comparable as well.

## 4. Conclusions and Outlook

In this study, a library of BADGE- and DEO-based epoxy resins, crosslinked with mixtures of IPDA and DAN, was investigated with special respect to the thermal conductivity. The majority of these polymer networks and nanocomposites exhibit thermal conductivities in the range of 0.1–0.2 W·m^−1^·K^−1^; only the DEO/DAN polymer network has a significantly increased (‘switchable’) thermal conductivity at elevated temperatures. The thermal conductivity increases from 0.229 to 0.437 W·m^−1^·K^−1^ in the temperature range from 90 to 120 °C; the glass-transition temperature of this resin is 98 °C. This significant increase of the thermal conductivity is argued to originate from π-π stacking of the bisphenyl-containing resin with a comparably high content of aliphatic/flexible segments. The increase of the thermal conductivity occurs only above the glass-transition temperature (at which segmental mobility within the polymer matrix increases) and only if silica nanoparticles, which can disrupt π-π stacking, are absent.

The surface energy of the DEO/DAN network is very comparable to that of the BADGE/DAN network. Correspondingly, if such DEO/DAN blends are employed in microelectronics and experience high temperature loads above the glass-transition point during operation, and the thermal conductivity increases above the glass-transition point, heat dissipation and decrease of the temperature load are accelerated. The dielectric and electric properties of such epoxy resins will be the subject of a further study; while commonly, and because of the mechanical properties, the application of polymers is favored at temperatures below their glass-transition point, these future studies will also focus on the fine-tuning of the glass-transition temperature by variation of the curing parameters.

## Figures and Tables

**Table 1:** Glass-transition temperatures of the bisphenol A diglycidyl ether (BADGE-) and 1,2,7,8-diepoxyoctane (DEO)-based epoxy-amine blends and composites with varying contents of diamine-based hardeners. IPDA, isophorone diamine; DAN, *o*-dianisidine.

Epoxy Type	Ratio DAN/IPDA (wt.-%/wt.-%)	Content SiO_2_ (wt.-%)	T_g_ (°C)
BADGE	0:100	0	154
BADGE	33:67	0	152
BADGE	67:33	0	149
BADGE	100:0	0	148
BADGE	0:100	5	152
BADGE	33:67	5	151
BADGE	67:33	5	146
BADGE	100:0	5	146
DEO	0:100	0	118
DEO	33:67	0	118
DEO	67:33	0	103
DEO	100:0	0	92
DEO	0:100	5	100
DEO	33:67	5	92
DEO	67:33	5	88
DEO	100:0	5	85

**Table 2:** Surface energy of the BADGE- and DEO-based epoxy-amine blends with varying contents of diamine-based hardeners.

Epoxy Type	Ratio DAN/IPDA	Content SiO_2_	Free Surface Energy	Polar Fraction	Disperse Fraction
	(wt.-%/wt.-%)	(wt.-%)	(mN·m^−1^)	(mN·m^−1^)	(mN·m^−1^)
BADGE	0:100	0	22.3	4.7	17.5
BADGE	33:67	0	36.3	4.2	32.0
BADGE	67:33	0	38.4	3.4	35.1
BADGE	100:0	0	39.5	1.1	38.3
DEO	0:100	0	25.0	5.0	19.5
DEO	33:67	0	32.7	4.3	28.4
DEO	67:33	0	34.8	3.2	31.6
DEO	100:0	0	41.3	0.1	41.2

## Data Availability

The data presented in this study are available on request from the corresponding author. The data are not yet publicly available due to the performance of on-going studies of the scientific findings that have been reported.
